# Supplementary material for: Significant Decrease in Childhood Obesity and Waist Circumference over 15 Years in Switzerland: A Repeated Cross-Sectional Study
Source: Nutrients. 2019 Aug 15;11(8):1922. doi: 10.3390/nu11081922 (PMC6722927; doi:10.3390/nu11081922)
Supplement: Supplementary file 1 [file nutrients-11-01922-s001.pdf]

Supplementary Table 1 Frequencies (%) or mean (±SD) values of answers given to the questions in self administered questionnaire by weight status group (based on CDC reference values)

	**Healthy weight**	**overweight**	**obese**
**Physically active for min 1 hour**			
≤ 1 day	65 (3.6%)	16 (7.0%)	13 (12.5%)
2-3 days	418 (23.2%)	84 (36.7%)	45 (43.3%)
4-5 days	711 (39.5%)	79 (34.5%)	30 (28.8%)
6 days	214 (11.9%)	17 (7.4 %)	8 (7.7%)
7 days	390 (21.7%)	33 (14.4%)	8 (7.7%)
**Softdrink consumption**			
≤ once/week	1115 (62.6%)	134 (59.8%)	58 (54.7%)
2-4 times/week	329 (18.5%)	43 (19.2%)	31 (29.2%)
5-6 times/week	70 (3.9%)	10 (4.5%)	3 (2.8%)
daily	267 (15%)	37 (16.5%)	14 (13.2%)
**Fruit- and vegetable juice consumption**			
< once per day	1347 (76.1%)	175 (78.1%)	69 (69%)
1-2 times/day	370 (20.9%))	43 (19.2%)	24 (24.0%)
3 and more/day	53 (3.0%)	6 (2.7%)	7 (7.0%)
**Fruit consumption**			
< once per day	584 (33.1%)	87 (39.0%)	44 (43.1%)
1-2 times/day	937 (53.1%)	100 (44.8%)	42 (41.2%)
3-4 times/day	203 (11.5%)	30 (13.5%)	10 (9.8%)
5 and more/day	42 (2.4%)	6 (2.7%)	6 (5.9%)
**Vegetable consumption**			
< once per day	556 (31.2%)	83 (37.6%)	44 (42.7%)
1-2 times/day	1089 (61.2%)	114 (51.6%)	51 (49.5%)
3-4 times/day	97 (5.4%)	18 (8.1%)	5 (4.9%)
5 and more/day	38 (2.1%)	6 (2.7%)	3 (2.9%)
**Fruit and vegetable consumption**			
< 1/day	360 (20.5%)	57 (25.8%)	28 (28.0%)
Once/day	239 (13.9%)	36 (16.3%)	15 (15.0%)
2 times/day	353 (20.1%)	37 (16.7%)	24 (24%)
3 times/day	325 (18.5%)	32 (14.5%)	8 (8%)
4 times/day	267 (15.2%)	23 (10.4%)	10 (10%)
≥5 times/day	211 (12%)	36 (16.3%)	15 (15%)
**Milk and dairy product consumption**			
≤ once/week	111 (6.2%)	19 (8.6%)	13 (12.4%)
2-4 times/week	189 (10.6%)	23 (10.4%)	10 (9.5%)
5-6 times/week	170 (9.6%)	25 (11.3%)	15 (14.3%)
once/day	570 (32.0%)	72 (32.6%)	33 (31.4%)
> once/day	740 (41.6%)	82 (37.1%)	34 (32.4%)
**Meat and fish consumption**			
≤ once/week	173 (9.7%)	21 (9.4%)	18 (17.8%)
2-4 times/week	628 (35.3%)	70 (31.4%)	26 (25.7%)
5-6 times/week	352 (19.8%)	46 (20.6%)	19 (18.8%)
daily	626 (35.2%)	86 (38.6%)	38 (37.6%)

Supplementary Table 1 (continued)

	Healthy weight	overweight	obese
**Do you normally eat breakfast**			
Yes, always	1341 (74%)	153 (67.7%)	63 (59.4%)
Only on weeknds/sometimes	290 (16.1%)	48 (21.2%)	28 (26.4%)
No, never	167 (9.3%)	25 (11.1%)	15 (14.2%)
**Screen time (total)**			
≤ 1h/day	955 (54.6%)	103 (46.2%)	36 (34.6%)
> 1 h – 2 h/day	542 (31.0%)	68 (30.5%)	34 (32.7%)
> 2 h – 3 h/day	159 (9.1%)	34 (15.2%)	21 (20.2%)
> 3 h/day	93 (5.3%)	18 (8.1%)	13 (12.5%)
**Parental origin**			
Both CH	1017 (56.9%)	101 (44.7%)	32 (30.8%)
CH and non-CH	354 (19.8%)	44 (19.5%)	23 (22.1%)
Both non-CH	416 (23.3%)	81 (35.8%)	49 (47.1%)
**Parental education**			
Low	76 (4.4%)	15 (7.0%)	17 (17.7%)
Medium	607 (35.3%)	102 (47.7%)	48 (50.0%)
High	1035 (60.2%)	97 (45.3%)	31 (32.3%)
**Diabetes**			
Yes	5* (0.3%)	0 (0%)	0 (0%)
No	1912 (99.7%)	241 (100%)	121 (100%)
**Asthma**			
Yes	55 (2.9%)	5 (2.1%)	1 (0.8%)
No	1862 (97.1%)	236 (97.9%)	120 (99.2%)
**Health perception (How do you judge your health?)**			
Very good	1436 (80.4%)	165 (74%)	73 (70.2%)
Good	327 (18.3%)	57 (25.6%)	28 (26.9%)
Reasonably good	20 (1.1%)	1 (0.4%)	3 (2.9%)
bad	3 (0.2%)	0 (0%)	0 (0%)
**Weight perception (Which description best matches you?)**			
Much too thin	25 (1.4%)	2 (0.9%)	0 (0%)
Too thin	271 (15.2%)	3 (1.3%)	3 (3.0%)
About right	1425 (80.1%)	168 (74.3%)	29 (28.7%)
Too heavy	53 (3.0%)	53 (23.5%)	59 (58.4%)
Much too heavy	4 (0.2%)	0 (0%)	10 (9.9%)
**Life satisfaction (1-10)**	8.7±1.4	8.7±1.3	8.5±1.6
**Sleep duration (h)**	10.49±0.61	10.37±0.65	10.25±0.68

* 3 cases were identified as type 1 while the other 2 are unknown.
